# Editorial: Neurological insights into communication and synchrony between others: what animal and human group communication can tell us

**DOI:** 10.3389/fnhum.2024.1415166

**Published:** 2024-05-02

**Authors:** Rachel C. Amey, Megan R. Warren

**Affiliations:** ^1^Department of Psychology, University of Maryland, Baltimore, MD, United States; ^2^Department of Biology and the Center for Translational Social Neuroscience, Emory University, Atlanta, GA, United States

**Keywords:** synchrony, human communication, EEG, vocal signaling, non-verbal communication

Communication is the cornerstone of human interaction, serving as the conduit through which ideas are exchanged, relationships are formed, and societies thrive. While we often think of communication using overt means, such as physical gestures and speaking to others, the intricacies of communication extend far beyond explicit communication, encompassing non-verbal cues and physiological actions that shape our understanding and interpretation of social interactions (Phutela, 2015; Symons et al., 2016). In fact, given the recent advances in artificial intelligence, interpersonal interaction can be extended to occur not between individuals, but instead between individuals and computer, further complicating the role of covert cues. Additionally, recent advancements in neurological research in both animal and human studies have shed light on the underlying mechanisms of non-verbal behavior, offering profound insights into the complexities of human group dynamics and interpersonal communication (e.g., Hirsch et al., 2018). Indeed, understanding human communication may require delving into the methods and findings of animal models, as animal models have offered significant insight into human psychopathology (Heller, 2016). Thus, in this Research Topic, we explore the intersection of human, artificial intelligence, and animal research regarding non-verbal communication. We highlight five seminal articles that contribute to our understanding of human communication utilizing diverse tools to understand these phenomena such as metacognition, animal models, and dyadic human interactions.

One article, *Neurophysiological and Emotional Influences on Team Communication and Metacognitive Cyber Situational Awareness During a Cyber Engineering Exercise*, demonstrates advancements in neurological technology and how they contribute to the understanding of human communication (Ask et al.). Researchers examine the realm of cyber operations, where human-to-human communication plays a pivotal role in achieving shared situational awareness for effective decision-making. Utilizing the Orient, Locate, Bridge (OLB) model, researchers investigate the neural correlates of metacognitive cyber situational awareness among cyber cadets. Their findings underscore the influence of neurophysiological and emotional factors on team communication, revealing the importance of vagal tone in shaping metacognitive judgments and mood. This study provides essential insights into the cognitive processes underlying effective communication in cyber defense, and more broadly, to hierarchical communication. Together, these results pave the way for innovative approaches to recruitment, education, and training in this critical domain.

A possible explanation for the relationship between vagal tone and communicative success in cyber operations is emotional state. *Functional Graph Contrastive Learning of Hyperscanning EEG Reveals Emotional Contagion Evoked by Stereotype-Based Stressors* shows how humans can transmit emotion to one another without being consciously aware (Huang et al.). Authors show how emotional contagion pervades dyadic interactions, shaping the dynamics of collaborative tasks and influencing performance outcomes. This article also employed EEG-based hyperscanning to unravel the neural mechanisms underlying emotional contagion in the context of stereotype-based stressors. Through functional graph contrastive learning (fGCL), researchers suggest the impact of emotional contagion on participants' neural activity patterns, revealing its substantial role in modulating performance trajectories. This study contributes valuable insights into the neural underpinnings of emotional dynamics in dyads, enriching our understanding of social interactions in diverse contexts.

Attention to not only one's emotional state, but one's physiological state, can impact communication and interpersonal synchrony, as shown in *Autonomic Synchrony Induced by Hyperscanning Interoception During Interpersonal Synchronization Tasks* (Balconi et al.). This article demonstrates that social interactions are inherently dynamic, characterized by reciprocal influences on emotional states and physiological rhythms. This work investigates the role of autonomic synchrony in dyadic interpersonal synchronization tasks, exploring the impact of interoceptive focus on physiological coherence. By employing hyperscanning techniques, researchers reveal higher synchrony between paired participants in heart rate variability (HRV), skin conductance level (SCL), and heart rate (HR) during tasks involving focused attention on one's own breathing. These findings highlight the interplay between interoception and interpersonal synchrony, offering new avenues for studying psychophysiological coherence in real-time social interactions. This research also shows how human communication and synchrony can be seen not only though explicit communication and neurological activity, but also cardiovascular responses.

Other research takes a more cellular approach, looking at mirror neuron systems (MNS; Bonini, 2017), which are essential in understanding the intentions and movements of others. In, *Effects of Avatar Shape and Motion on Mirror Neuron System Activity*, researchers explored the role of the MNS in perceiving humanness in avatars, shedding light on how avatar characteristics impact neural activity (Miyamoto et al.). Application of electroencephalogram (EEG) analysis demonstrated activation of the MNS in response to human-like avatar shapes and motions, highlighting the importance of considering both visual and kinematic cues in avatar design and interpreting the intentions of others through physical movement. These findings offer valuable insights for enhancing inter-avatar communication and fostering a sense of social presence in virtual environments. Further, they demonstrate how understanding human communication in humans is an automatic process that can extend beyond assessing other humans, even down to the neuronal level.

Animal research provides further evidence for the influence of non-verbal communication. In *Listening to Your Partner: Serotonin Increases Male Responsiveness to Female Vocal Signals in Mice*, researchers explore how the context surrounding vocal communication can significantly influence the perception of vocal signals (Hood and Hurley). Specifically, authors examined serotonin's role in modulating behavioral responses to vocal signals in mice. By manipulating serotonin levels systemically and locally in the inferior colliculus (IC), researchers uncover the nuanced effects of serotonin on vocal behavior, highlighting the neurotransmitter's role in modulating male responsiveness to female vocal signals. These findings underscore the importance of considering neurotransmitter systems in understanding the mechanisms of context-dependent communication.

In conclusion, the articles presented in this Research Topic offer a multifaceted exploration of non-verbal communication from neurological perspectives, spanning human and animal research domains. From cyber defense decision-making to avatar design in virtual environments, interpersonal synchrony in social interactions, emotional contagion in dyadic tasks, and autonomic synchrony to serotonergic modulation of vocal perception, these studies illuminate the diverse facets of non-verbal behavior and its underpinnings. By integrating insights from human and animal models, we can deepen our understanding of communication dynamics and pave the way for future advancements in understanding an innate human behavior.

## Author contributions

RA: Conceptualization, Writing – original draft. MW: Conceptualization, Writing – review & editing.
